# Chromatin Regulation and Genome Maintenance by Mammalian SIRT7

**DOI:** 10.1093/geroni/igaa057.2653

**Published:** 2020-12-16

**Authors:** Joonseok Cho

**Affiliations:** Stanford School of Medicine, Stanford, California, United States

## Abstract

Members of the Sirtuin family of enzymes are important regulators of genomic stability, stress responses, and metabolic programs that impact on human physiology, aging, and age-related disease processes. We previously showed that the mammalian Sirtuins SIRT6 and SIRT7 have high-selectivity histone deacetylase activities at chromatin, and inactivation of SIRT6 or SIRT7 results in dysregulated histone acetylation states and gene expression programs, with pathological consequences at the cellular and whole organism levels. Recently, we have been exploring novel functions of SIRT6 and SIRT7 in silencing of heterochromatic regions of the genome, the deregulation of which has been linked to aging and cancer biology. We found that pericentric heterochromatin silencing by SIRT6 prevents acute cellular senescence that is triggered by pathologic pericentric transcripts. We also uncovered a second novel trigger of human cellular senescence, ribosomal DNA instability in nucleoli, and we showed that SIRT7 guards against senescence induced by this instability. In our studies, a long-term focus has been identifying substrates of SIRT6 and SIRT7 and their roles in aging and disease pathways. In new work, are studying a novel physiologic substrate of SIRT7 at chromatin, H3K36Ac, and we are characterizing the genomic landscape of this SIRT7-dependent deacetylation target, and its downstream chromatin and nuclear signaling mechanisms. We will also discuss mechanistic insights into the functions of SIRT6 and SIRT7 from new proteomic, cellular, and mouse model studies.

